# Empowerment: Freud, Canguilhem and Lacan on the ideal of health promotion

**DOI:** 10.1007/s11019-023-10145-z

**Published:** 2023-04-27

**Authors:** Bas de Boer, Ciano Aydin

**Affiliations:** Philosophy Group, University of Twente, Enschede, The Netherlands

**Keywords:** Empowerment, Health promotion, Sublimation, Canguilhem, Psychoanalysis

## Abstract

Empowerment is a prominent ideal in health promotion. However, the exact meaning of this ideal is often not made explicit. In this paper, we outline an account of empowerment grounded in the human capacity to adapt and adjust to environmental and societal norms without being completely determined by those norms. Our account reveals a tension at the heart of empowerment between (a) the ability of self-governance and (b) the need to adapt and adjust to environmental and societal norms. We address this tension by drawing from the work of Freud, Canguilhem, and Lacan. First, we clarify through a discussion of Freud’s notion of sublimation that it is difficult to assess empowerment independent of any social valuations, but also that it is no less problematic to make it dependent on social valuations alone. Second, we draw from the work of Canguilhem to show how empowerment can be understood in terms of the individual’s capacity to tolerate the aggressions of a multiplicity of environments. Third, using Lacan, we show how empowerment requires incorporation of social and symbolic norms, without necessarily rendering ourselves a mere product of these norms. Finally, we demonstrate how the views of these authors can complement one another, resulting in a more sophisticated understanding of empowerment.

## Introduction

For several decades now, health promotion has been a central objective of the World Health Organization (WHO) and individual governmental bodies alike. The WHO defines health promotion as “the process of enabling people to increase control over, and to improve their health” (WHO 2021, 4).^1^ Health promotion is said to contribute to empowerment, which is defined as “a process through which people gain greater control over decisions and actions affecting their health” (Ibid., 14). Hence, empowerment allegedly helps people to gain control over many factors in their lives that are relevant to their health, but which currently are not or cannot be (immediately) utilized. Health promotion is thought to contribute to the realization of this ideal (Cyril et al. 2016). However, what is exactly meant by the process referred to in the WHO definition of “empowerment” remains unclear, which also makes it difficult to understand how health promotion helps empowering individuals.

It has been stressed that empowerment presupposes a form of self-governance (e.g., Agner and Braun 2018, Tengland 2016). However, since humans are always situated in an environment that imposes norms on them, they are never completely free in how to govern themselves, but also need to adapt to certain social and environmental norms. These norms are depended on one’s own bodily condition in relation to the environment (e.g., I cannot walk through a wall, or I will get ill or die when being exposed to extreme temperatures), are socially constituted (e.g., a good parent should behave in this way) or are a mixture of both (e.g., given your ability to take care of your material body, you should aim to reach a certain age). As the latter type of norms indicate, biomedicine and health promotion have normative import and therefore play an important role in determining what is sick, normal and healthy (Hancock 2018; Harvey 2010). Complete coincidence with a particular (set of) norm(s) cannot be considered to be a form of empowerment, as this would imply that someone is governed by something external rather than engages in *self-governance*. At the same time, one’s ability to set one’s own norms is always limited by things that are beyond one’s control, as it is impossible to completely detach oneself from one’s biological constitution or societal context.

Accordingly, empowerment involves a tension between self-governance and adaption. In this paper, we address this tension by drawing from the work of Sigmund Freud, Georges Canguilhem, and Jacques Lacan. Earlier, the work of these authors has been mobilized to critique the hegemony of biomedicine when it comes to determining health and illness (e.g., Zwart 1998), and to point to the fact that the curative activity of medicine should carve out a space for patients to come to terms with health and illness in ways that are not completely determined by biomedical discourse (e.g., Mordacci 1998). In line with such analyses, we take it that these authors are relevant to the discussion of empowerment because they all recognize, albeit in different ways, that self-governance crucially involves the capacity to adapt and adjust to the norms that are imposed on them without being completely determined by those norms. Therefore, the work of Freud, Canguilhem and Lacan can be a starting-point for analyzing how people need not merely to adapt to the norms that health promotion imposes on their existence but can integrate health promotion in the project of creating its their norms.

Our paper is structured as follows: First, we briefly introduce how health promotion intends to contribute to empowerment. Second, we clarify through a discussion of Freud’s notion of sublimation that it is difficult to assess empowerment independent of any social valuations, but also indicate that it is no less problematic to make empowerment dependent on social valuations alone. Second, we draw from the work of Canguilhem to show how empowerment can be understood in terms of the individual’s capacity to tolerate the aggressions of a multiplicity of environments. Third, using Lacan, we show how empowerment requires incorporation of social and symbolic norms, without necessarily rendering ourselves a mere product of these norms. Finally, we demonstrate how the views of these authors can complement one another, resulting in a more sophisticated understanding of empowerment.

## Health promotion and empowerment

Health promotion aims to increase the ability of individuals to take control and responsibility for their own health. This, so it is maintained, has various upsides, such as a decrease in the amount of hospital visits, or a general increase in the well-being of a population. Part of the success of health promotion relies on the prevention of future diseases by changing lifestyle choices of individuals. Currently, this success seems to be increasingly dependent on the active participation of people that collect health-related data about their own lives (e.g., European Commision 2018). This marks a difference with earlier practices contributing to the prevention of diseases such as vaccination programs (although this has become very topical again) or measures for ensuring clean drinking water, that either involve environmental interventions or interventions that are not dependent on personalized and customized data (Mayes and Armistead 2013, p.691). Another distinctive feature of current forms of health promotion is that its success is dependent on the willingness of people to voluntarily change their behavior and/or make lifestyle changes that are beneficial for their health; for example, abstaining from certain behaviors (e.g., overeating, alcohol use), or more frequently engaging in others (e.g., physical activity).

Because of the abovementioned features, it is often said that health promotion contributes to empowerment (Agner and Braun 2018; Cyril et al. 2016; Halvorsen et al. 2020). Philosophical analyses of empowerment highlight that, although the notion of empowerment is generally not well-defined, the term is often used in relation to the abilities of self-control and self-governance (e.g., Morley and Floridi 2020; Tengland 2007, 2008, 2016). Tengland intends to provide conceptual clarification on the nature of empowerment by distinguishing between empowerment as a goal of health promotion and empowerment as a process that health promotion should engage in. He argues that empowerment can refer to (a) the desired outcome of health promotion when it contributes to an increase in control and knowledge about those aspects of someone’s life relevant to their health, and (b) a decrease in the power of healthcare professional over the types of decisions that individuals make in a medical context (Tengland 2008). Health promotion, then, allegedly contributes to empowerment because it increases the decision-making capacities of individuals or groups through increased knowledge of and control over relevant aspects of their own lives (Tengland 2016, p.34). Additionally, “empowerment” can refer to the implementation of those measures that help people increase their capacity to set and pursue their own goals, rather than goals predetermined by someone else, such as the government, society, and health professionals.

The ideal of empowerment in health promotion points to changing both the socio-economic circumstances in which people live and the development of skills that increase their ability of self-governance. Therefore, empowerment also requires sufficient knowledge about what one’s decisions entail, the societal and political context in which those decisions are executed, and the motivation to embark on them (Tengland 2007, p.199). For example, if a person decides to become a parent, raise two children, and pursue a career at the same time, they are allegedly more empowered if they have more control over the determinants that contribute to achieving this. This requires for example (financial) access to (day) care facilities for children, awareness that such care facilities exist, adequate housing to raise children, and knowledge about which behavior to avoid when being pregnant, as well as the ability to act upon this.

Additionally, it has been pointed out recently that the notion of empowerment in current health promotion practices does no longer coincide with the ideal of patient empowerment, as many people that are candidates for “being empowered”—for instance through the use of mobile health (mHealth) devices or self-tracking technologies—are not necessarily patients yet (Kapeller and Loosman 2023). As a result, the notion of empowerment as well as the practice of health promotion become increasingly disconnected from professional care settings and the biomedical sciences more generally. In response to this development, Morley and Floridi (2020) have argued that the framing of many mHealth devices in terms of them being empowering is misleading because it is unclear how they contribute to empowerment in a more strictly medical sense.

More generally, the empowerment approach can be criticized by pointing to the fact that it seems to be implicitly assumed that empowered people are generally good at taking care of their own health and well-being, whereas it is far from certain that self-governance is an ability that secures healthy behavior in the long run, and therefore potentially runs counter to the aims of health promotion. Even when people are empowered, it seems, they may choose to engage in risky activities (e.g., mountain climbing) and/or behave in ways that are or may be harmful to their health (e.g., remaining to smoke, consuming alcohol and drugs). Two possible responses to this criticism are: (1) Empowerment has not yet been realized when a large number of individuals remains to live a life that is detrimental to their health, such that current interventions for empowerment are to be judged as thus far unsuccessful but potentially successful in the longer run (e.g., Braunack-Mayer and Louise 2008); (2) To the extent that health is to be considered a form of empowerment, a broader understanding of health is needed than one which exclusively understands health in terms of the absence of disease, the avoidance of risk, or the leading of a life that fully conforms to externally established health standards, including those set by healthcare professionals (Tengland 2016). The latter issue seems especially relevant given the changing target of empowerment practices in health promotion through the development of mHealth devices and/or self-tracking technologies.

So, on the one hand, to the extent that health promotion is trying to contribute to empowerment, it must somehow be able to distinguish cases of empowerment from other cases. On the other hand, it seems that a narrow biomedical understanding of health does not offer the tools to make this distinction. In the next sections, we outline an account of empowerment that intends to address these issues, starting with a discussion of Freud’s notion of sublimation.

## Freud and the problem of identifying sublimation

Although Freud might not be the first to come to mind when discussing if and how health promotion can be said to empower people, his thought is relevant when linking empowerment to the abilities of self-control and self-governance. In this respect, Freud thematizes the tension between individual urges and preferences on the one hand and societal norms on the other. For Freud (especially in his earlier works), being healthy involves the ability to redirect one’s sexual drives, which requires a form of self-governance that he calls *sublimation*. He relates sublimation to “deflecting the sexual instinctual forces away from their sexual aim to higher cultural aims” (Freud 1981, p.1956). These forces have a broader scope than sex in the narrow sense of the word and refer to basic urges that demand immediate gratification. Sublimation manifests itself in behavior or activity that can be considered socially or culturally valuable, which Freud used as a criterion to distinguish between health (sublimation) and disease.

Freud’s definition of sublimation might appear straightforward but has prompted a great deal of discussion and has been a source of a lot of confusion (Laplanche and Pontalis 1973, p.433). Freud’s repression theory indicates that drives that have been repressed can manifest themselves as substitute formations in various types of behavior. However, his account of sublimation does not seem to enable separating those substitute formations, those instances of the “return of the repressed, (Freud 1981, p.2215, p.2984)” which are (neurotic) symptoms, from those that are genuine sublimations (Gemes 2009). For example, someone who washes her hands 100 times a day may have redirected a sexual aim (for example excessive masturbation) to a nonsexual one, but that could hardly be considered a case of successful sublimation, as also Freud would acknowledge. Even more importantly, many cases—such as very frequent hand washing—seem difficult to assess in terms of social valuation. At the same time, they also cannot be completely separated from it; or at least from how others think about it, even in the absence of a clear social norm. Frequent hand washing can be a social habit or even a duty, as was very clearly the case during the COVID-19 pandemic. So on the basis of Freud’s definition of sublimation it remains unclear when a redirecting of primal urges is successful and, hence, can be called healthy.

This difficulty might be partly explained by Freud’s famous recognition that it is impossible to sharply and unambiguously distinguish the normal from the pathological as binary categories: “We no longer think that health and illness, normal and neurotic people, are to be sharply distinguished from each other, and that neurotic traits must necessarily be taken as proofs of a general inferiority” (Freud 1981, p.2300). Nevertheless, Freud’s psychoanalytic practice could not do without making some distinction between the normal and the pathological.

Other critics of Freud’s view of sublimation have attempted to provide a strict psychoanalytic account that makes it possible to distinguish between health and illness without relying on social valuations. For example, Fenichel tried to do so on the basis of the distinction between sublimation as a successful defense and repression as an unsuccessful defense (Fenichel 1945). Although the distinction between repression and sublimation is not always clear in Freud, there are passages that strictly distinguish the two, which Fenichel seems to take as lead: “Premature repression makes the sublimation of the repressed instinct impossible; when the repression is lifted, the path to sublimation becomes free once more” (Freud 1981, p.2238). Fenichel discusses three differences between repression and sublimation. First, he indicates that in sublimation, “the original impulse vanishes because its energy is withdrawn in favour of the cathexis of its substitute” (Fenichel 1945, p.141). Second, he argues that sublimated impulses “find their outlet,” whereas repressed impulses do not (Ibid., p.141). Third, he points out that in sublimation, as opposed to neurotic substitute gratifications, there is a desexualization (Ibid., p.142).

Gemes tries to illustrate on the basis of Freud’s discussion of the Leonardo (da Vinci) case that Fenichel’s distinction between (unsuccessful) repression and (successful) sublimation cannot be sustained (Gemes 2009, p.45). He argues that one could hardly say that Leonardo’s original homosexual impulses had vanished, since they are expressed in many of his activities, such as his drawings of perfect idealized male bodies. This observation also challenges Fenichel’s idea of desexualization as a marker that separates sublimations from pathological symptoms. We have seen that also the example of hand washing displays the unsustainability of this distinction, which makes Fenichel’s as well as Freud’s distinction between pathology and sublimation, in terms of the distinction between sexualized and nonsexualized expression, untenable. Both sublimations and pathological symptoms could appear in both sexualized and nonsexualized forms.

It seems to be difficult to understand sublimation in strictly psychoanalytical terms independent of a social context and social valuations. At the same time, making sublimation dependent on what a particular society happens to find acceptable is, as Laplanche and Pontalis indicate, also not without problems, since it could lead to repression and arbitrariness: “Should the fact that activities described as sublimated in a given culture are accorded particularly high social esteem be taken as a defining characteristic of sublimation?” (Laplanche and Pontalis 1973, p.433).

Clearly, throughout his work, Freud explores different ways of distinguishing between health and illness (Tran The 2018). It must be clear that we focus here on how he makes this distinction in the context of his notion of sublimation. Moreover, the above discussion is not intended as an attempt to debunk nor definitively clarify what Freud means by sublimation. Rather, our discussion serves to illustrate that it is not a coincidence that reflection on the meaning of “sublimation” in Freud’s work highlights a tension between drives and social valuation, a tension that needs to be taken into account in the concept of empowerment. The tension between “unregenerate instincts and overbearing culture” lies at the heart of Freud’s psychoanalysis: “Since the individual can neither extirpate his instincts nor wholly reject the demands of society, his character expresses the way in which he organizes and appeases the conflict between the two” (Rieff 1965, p.28). Freud does not seem to render a final resolution between the two forces possible but does seem to consider social valuation credible as a criterion; while acknowledging that culture always remains potentially repressive and urges always lurk beneath the surface, ready to disrupt socially accepted behavior, his criterion for health ultimately seems to lie in alignment with the values espoused in his day (Aydin 2021, p.213).

Freud’s work thus reveals a tension between the abilities of self-governance and adaption. Sublimation makes it possible to direct one’s power from the instant gratification of raw urges to the development or execution of socially valued projects. Clearly, sublimation could potentially provide a particular interpretation of self-legislation and empowerment. However, it remains unclear how this interpretation of sublimation enables developing a *critical relation* with socially valued norms. In other words, what constitutes successful sublimation in the tension between what an individual is and wants, on the one hand, and what the environment or a society allows and demands, on the other?

In the next two sections we explore this question further and outline how empowerment arises in the conflict between individual and environment. We do so by first introducing Canguilhem’s approach to health that grounds it in the organism’s biological non-indifference to the environment, and second by drawing on the work of Lacan who argues that sublimation can only be understood in relation to a given symbolic order.

## Georges Canguilhem’s notion of biological normativity

As can be inferred from the above discussion, any idea of what constitutes *critical* self-governance implies to consider an individual in relation to the environment in which it finds itself. When medicine attempts to empower people by being concerned with the behavior and lifestyle choices of individuals, therefore, empowerment cannot solely understood in terms of physiological or molecular processes inside one’s body. Moreover, as we saw in our discussion of Freud’s notion of sublimation, there is also a potential tension between the goals society sets for living a healthy life and the idiosyncratic preferences of individuals and their conceptions of what constitutes a good life.

In this section, we show, drawing from the work of Georges Canguilhem, that the criterion by which a distinction between health and illness is made need not lie in a source external to the organism—as Freud would eventually have it—but can be located in the qualitatively different relationships between organism and environment. Although Canguilhem’s work focuses on health and nowhere explicitly speaks about empowerment, his work on health is relevant to our discussion as he discusses health in terms of the organism’s capacities of norm-setting and self-regulation. For Canguilhem, as we will see, health consists not so much in a particular (observable) state but rather in particular capacities that enable the organism to relate to a multiplicity of environments without being determined by the norms of a single environment. For this reason, we take Canguilhem’s account of health in this paper to be in fact an account of empowerment as it addresses the same tension between self-governance and adaption.

### Life’s non-indifference to the environment

Canguilhem wrote extensively on the dynamic relationships between organisms and their environment. He maintains that these relationships are characterized by what he calls a *biological normativity* on the side of the organism: organisms are non-indifferent to their environment in the sense that they react more or less spontaneously to those aspects of the environment that obstruct their preservation. They do so by imposing new norms on themselves or on the environment, which result into a modification of either the organism’s conduct or the environment (Canguilhem 1991, p.126: see also Sholl 2016). For Canguilhem, biological normativity is a basic characteristic of life: life, so he maintains, “is far removed from […] an indifference to the conditions which are made for it; life is polarity” (Canguilhem 1991, p.128). In this sense, life is a norm-setting activity in constant struggle with that what prevents the realization of key biological values (e.g., food, survival, harm avoidance), and, hence, is inherently future-oriented (Rand 2011; Trnka 2003).

In line with this idea, the healthy organism expresses a particular form of normativity that structures how it relates to its environment, and which is constitutive of the extent to which it can adapt to and adjust the environment (Canguilhem 1991, p.77). Health must be understood “the possibility of transcending the norm, which defines the momentary normal, the possibility of tolerating infractions of the habitual norm and instituting new norms in new situations” (Ibid., p.115). These norms are not equivalent to mechanistic functions that stand in a causal relationship with certain environmental mechanisms. Such an understanding of norms mistakenly takes the environment in which organisms live to be a (fixed) *physical* environment; as an already constituted fact. However, biologically speaking, “the organism is not thrown into an environment to which he must submit, but he structures his environment at the same time that he develops his capacities as an organism” (Ibid., p.284). Hence, the environment to which the organism relates is no theoretical abstraction remaining unchanged over time but is instead relative to the organism’s ability to modify it and/or adapt to it.

Since organisms continually move through different environments, they are in continuous need to exercise their biological normativity in different circumstances. Health, then, for Canguilhem, is an expression of the organism’s capacity to cope with the multiplicity of environments that the organism inevitably faces, each asking for a certain response: it “is a margin of tolerance for the inconstancies of the environment” (Ibid., p.197). For example, being healthy involves the ability to tolerate weather changes (or changes in climate when moving to another country), such that the organism does not experience such changes as a threat to its capacity of organization. Or the organism might employ certain technical means to build houses for shelter, thereby structuring the environment it needs to adapt to. An extreme example of the reverse of the capacity of organization would be a fetus only capable of remaining to live in an incubator, and which is too weak to be exposed to the environment outside of it and passes away in this very exposal.

In health, the organism can execute its powers without actively being constrained by the environment in which it lives. In this case “life [is] being lived in the silence of the organs,” such that the healthy individual is someone “who adapts silently to his task, who lives the truth of his existence in the relative freedom of his choices” (Canguilhem 2012, p.49). In illness, by contrast, an organism experiences active transformation in its relation to the environment that makes that it “feels inferior to the task which the new situation imposes on him” (Canguilhem 1991, p.182). The sick organism experiences a qualitative transformation because a new *norm* is instituted through which an organism evaluates its powers and limits when relating to a given environment differently. In illness, the organism does not cease to be normative, but instead institutes a new norm, making that it feels unfit to “tolerate and compensate for the aggressions of the environment” (Canguilhem 2012, p.49). Examples of such aggressions are viruses, or the weather changes mentioned above. It is this new norm constituted in relation to such aggressions that, for Canguilhem, marks a criterion for making the distinction between health and illness, because it marks a new way of being non-indifferent to the environment.

### Self-regulation and the difference between illness and anomaly

According to Canguilhem, for the human organism, being healthy implies not only to live life “in the silence of the organs,” but also “in the discretion of social relations”. After all, “[i]f I say […] that I am unwell, people want to know how and why; they wonder or ask me whether I am registered with social security” (Ibid., pp.49–50). This indicates that for Canguilhem, being healthy also involves not being burdened with the specific social and institutional norms that helps constituting what is understood to be a “healthy life” in a given society.

Yet, in the case of health promotion, the norms about what constitutes a healthy life become increasingly important when evaluating the life choices of individuals. This is because health promotion is no longer solely concerned about curing people, but focuses on taking measures to prevent people from becoming sick in the future, even when these people might (still) feel healthy in the present. Therefore, health promotion shapes what are taken to be forms of appropriate self-governance (Hancock 2018). This prompts the question of the extent to which social norms, including the ones put forward in health promotion (e.g., a certain average lifespan in a given population), play a role in shaping what is experienced as a healthy norm.

Addressing this question requires considering how Canguilhem distinguishes between how organisms and societies are organized. In making a distinction between the two, he critically reacts to philosophers and biologists that compare the organization of society to the organization of organisms (e.g., August Comte, or Walter B. Cannon). According to Canguilhem, this comparison is fundamentally mistaken, because it fails to distinguish between two different forms of regulation: a *self*-regulation, and a form of regulation that is external to the existence of something, where the former applies to organisms and the latter to societies.

The origin of organismic regulation is to be found in the organism’s non-indifference to the environment that manifests in the organism’s activity of norm-setting, which Canguilhem calls, as indicated before, biological normativity. Organisms “live as a whole and [are] not […] able to live except as a whole. This is made possible by the existence […] of a set of apparatuses or mechanisms of regulation whose effect consists precisely […] in the persistence of the organism as a whole” (Canguilhem 2012, p.72). Societies, by contrast, are “always out of order, because [they] are deprived of [a] specific apparatus of self-regulation” (Ibid., p.77). As a result, it is continuously needed to agree collectively in accordance with which norms and ideals a given society should be regulated. Societies, then, are always characterized by a certain incompleteness because their functioning is dependent on something external, such that they can never be a completely organized whole. Relying on social valuations as a way of distinguishing health from illness therefore appears arbitrary: “To define abnormality in terms of social maladaptation is more or less to accept the idea that the individual must subscribe to the fact of [a given] society” (Canguilhem 1991, pp.282–283). For this reason, Canguilhem, in contrast with Freud’s account of sublimation discussed earlier, does not conceive of “healthy organization” in terms of its coincidence with socially valued ends.

The difference in how societies and organisms are organized has consequences for the demarcation between health and illness. This can be further clarified through the work of the Gestalt psychologist Kurt Goldstein whose work greatly inspired Canguilhem’s. Goldstein makes a distinction between *illness* and *anomaly*. He maintains that whereas concepts like health and illness are norms of living for the concrete individual, the notion of anomaly points to an external observation of an organism that compares it against the species that it belongs and/or the specific social communities (e.g., nationality, age-group, neighborhood, level of education) that it is part of (Goldstein 1995, p.344). The concept of anomaly thus suggest that it is possible to make a quantitative distinction between the normal and the pathological by comparing an individual to relevant aspects of the population to which it “belongs”.

According to Goldstein and Canguilhem, however, such an interpretation fails to notice that health and illness must be considered relative to how organisms regulate themselves and exercise their norm-setting capacities. Different ways of self-regulating, as we saw, constitute the experience of being fit or unfit to exercise one’s powers on the environment. It is this qualitative experience that, for Canguilhem, functions as a demarcation criterion between health and illness. While it is perfectly possible that the social norms and valuations shape the experience of illness, they are in themselves necessary nor sufficient conditions for *demarcating* between health and illness. This demarcation takes place on the level of the organism that might or might not experience its relations with the environment as pathological (i.e., as instantiating a qualitative transformation through which it feels unable to tolerate and compensate for the aggressions of the environment). This experience is organized around the organism’s ability to remain to have a degree of individuality, which makes it possible that the organism remains capable of having a certain stability in relation to the variety of environments it faces and does not completely integrate with the social norms and averages but is capable of organizing itself in relation to those (Gayon 1998). In both cases, it is key for the organism to maintain a certain degree of individuality that makes norm-setting possible and prevents it from completely merging with one particular environment.

Generally, the discussion of Canguilhem reveals that health can be understood in terms of the capacities of norm-setting and self-regulation. Since these capacities are taken to be central to empowerment, Canguilhem’s insights into health can be extrapolated to the context of empowerment. Now, what does Canguilhem’s approach to health reveal about the ideal of empowerment in health promotion? First, it shows that empowerment should not concern the organism in isolation, but always in terms of its relation with the environment. Second, it indicates that empowerment can be fruitfully understood as the organism’s capability to tolerate the aggressions of different environments. Whereas Canguilhem tends to understand such aggressions mostly to be biological dangers, the next section on Lacan will show that such aggressions can equally have a social origin – and that the two are difficult to entangle. Third, insofar as empowerment involves the promotion of the ability of self-governance, it cannot fall back onto a certain societal average that is idealized and marked as an indicator of health or empowerment. Instead, it refers to the cultivation of the organism’s capacity of self-regulation that makes it possible to maintain its individuality in its relation with the environment, and to not merge completely with any given environment. Empowerment, then, is a mark of the capacity to cope with particular environments without being a sheer product of those environments.

## Lacanian sublimation: empowerment as transgression within a symbolic order

In the previous sections, empowerment has been interpreted as a mark of successful or unsuccessful coping of individuals in respect to their environment. With Freud, we have seen that empowerment is difficult to interpret independent and outside of a sociocultural context. Through a discussion of Canguilhem’s work, it became clear that empowerment can be understood in terms of the experience of being able to tolerate the aggressions of different environments. In this section, we intend to clarify how this experience is fueled by sociocultural norms. We do so by discussing Lacan’s appropriation of the Freudian notion of sublimation that results in a more sophisticated conception of both environment and individual, as well as of the tension and interaction between the two.

Partly continuing Freud’s line of thought, the tension between drive and culture is expressed more or less consistently throughout Lacan’s work in his account of the three categories or dimensions of otherness: the Real, the Imaginary and the Symbolic. The Imaginary and the Symbolic constitute for Lacan the inter- and trans-subjective world that enables the individual to engage with other people and hook into a general language and reason. The Imaginary and Symbolic are related but do not coincide: the Imaginary is central to Lacan’s account(s) of ego formation and manifests itself in dyadic relations (such as identifying with ones mirror image or with a parent’s profession), whereas the Symbolic constitutes triadic relations by introducing, besides an intersubjective relation, a trans-subjective symbolic order that normatively regulates the relations between particular beings and society (Lacan 2006, p.44, p.365, p.388). By subjecting itself to laws and restrictions that control and regulate its desires, interactions with others and the rules of communication, the self enters the Symbolic order and becomes a subject. Since biomedicine is part of the symbolic order, it is also constitutive for imposing laws and restriction on what constitutes a healthy subject (Zwart 2016).

The Real is a border-category that is difficult to define due to the irreversible entrance to the Imaginary and Symbolic order that has shaped and is shaping the subject. It refers to a primordial state that precedes the establishment of the symbolic order. Because what falls outside the Imaginary and Symbolic order is incognizable, it can only be denoted as a loss, a void or a lack. However, it leaves its traces as a danger and a possibility that can disrupt and undermine any symbolic order. For Lacan, it is by virtue of the Real that an individual cannot be completely absorbed by the general, including ethical, political, social and technological rules and regulations. The Real is that what I cannot grasp, generalize and compare, what resists being expressed in language and symbols. And it is by virtue of the Real that I can be different, that is, a singular person.^2^ A person is characterized by some-Thing that is not generalizable and cannot be completely reduced to general structures and features (Visker 2005, p.438). Besides the projections of other people and the symbolic order that imparts its rules and regulations on me, my subjectivity is also characterized by a difference or singularity that can never be completely absorbed and nullified by those ideal images, projections and regulations.

This tension between the Imaginary/Symbolic and the Real can also be illustrated by contemporary developments in medicine. For example, a digital twin that dynamically reflects molecular, physiological and lifestyle changes over time always misses out on some-Thing—a residue—that cannot be articulated, nor appropriated (de Boer 2020, pp.408–409); at the same time, what is left out makes me the different, unique, singular person that I am. This residue remains, as Lacan has pointed out repeatedly, inaccessible, also for the self in question (Lacan 1997, p.52, p.54). This is for example visible in contemporary practices such as self-tracking and neurofeedback in which the efforts and difficulties that are needed to relate those images to one’s existence become clearly visible (Brenninkmeijer 2013; Vegter et al. 2021). These mirror-like images, and every other possible image of me that I can envision, are incapable of capturing what makes me “me,” not only for others but also for myself. Subjectivity therefore seems to be characterized by something that escapes any order, be it biological, technology, or symbolic. In the words of Lacan: “[by] something that cannot or refuses to integrate into a functional totality” (Lacan 2006, p.78). In contrast with Canguilhem, who considers (human) organisms to be functionally integrated wholes, Lacan maintains that an inappropriable residue, as a mark of the Real, is the dimension that makes a human a particular, singular person and ensures plurality and novelty; the singular person is not a whole but a whole with a hole, a split subject. It is also this dimension of residue that makes the subject irreplaceable and, hence, gives it dignity. In addition, a proper positioning with respect to this residue is a condition for a “healthy” life (Lacan 1997, p.122, p.118; Moyaert 2011, p.250).

In contrast to Freud (Lacan would probably say ‘in addition’), not only is there an interaction between individuals and the environment but individuals become individuals by virtue of their interaction with their environment. A human being is born in a world and is bound to a language that it has not chosen, nor determined, and continuously attempts to cope both with its unsettled subjectivity due to the fact that it is formed by the symbolic order but does not fully coincide with it. The symbolic and technological order and the ideal images that we pursue are necessary conditions for forming a unified, coherent and social self, but are at the same time obstacles that prevent the subject from reaching the singularity that it seeks. This is not only because the subject has been shaped by them or cannot meet the demands imparted on it, but also because it can never fully appropriate its own singularity.

In *The Ethics of Psychoanalysis*, Lacan further elaborates on this tension and provides some normative grounding for what sublimation could be. Lacan, using his own idiosyncratic idiom, defines “sublimation” as the process that “elevates an object to the dignity of the Thing” (Lacan 1997, p.110). Lacan attempts to recognize both the value of a symbolic, moral law and the desire to undermine it. In fact, both are strongly interrelated: order produces the perverse desire to undermine order. The law regulates but also offsets desire in such a way as to increase the sense of temptation and going beyond a pre-established legal and moral order.

Now the difference between Freud’s and Lacan’s notion of sublimation becomes clearer. In line with Freud, Lacan conceives sublimation as a “form of satisfaction of the *Triebe*” (Ibid., p.110). However, he does not conceive of sublimation as a redirection of the libido toward a nonsexual object, as providing a socially accepted substitute for sexual gratification. This would imply the existence of a stable subject or ego that needs to be provided with the adequate means to control the Real, such that it can make beneficial use of its inclinations. For that matter, Lacan thinks that Freud never meant this either but rather was propagated by ego psychologists who misunderstood him. Be that as it may, sublimation for Lacan does not relate to a sublime moral beauty that supports a particular ethical framework or the assimilation of desires to social demands, but rather concerns symbolically organizing our drives in such a way that the Real, which generates a kind of emptiness or void, is not evaded but enabled to exert its influence. In organizing and satisfying our drives, we need to attempt to revisit the original object of desire and, at the same time, to somehow affirm it as the empty center (and lack) that fuels the desire to never (want to) coincide with any fixed environment. Sublimation is symbolic creation fueled by the Real and pushed to its limits, beyond (traditional or contemporary) moral constrains (Ibid., pp.115–127). In short, sublimation is the creation of the singular within the symbolic.

How can this Lacanian notion of sublimation contribute to the further development of a more apt notion of empowerment as a mark of the relation between the individual and its environment? First of all, we need to acknowledge that an individual is already subject to and is even shaped by the laws, mores, customs and restrictions of an Imaginary and Symbolic Order that controls and regulates its drives, desires and conduct. Empowerment, therefore, cannot only be the result of the particular qualities of an individual, but always involves being co-shaped by the images imparted by others and the laws and morals imposed, which make it possible to individuate and become a subject in the first place. One’s wishes and desires are to a great extent a repository for the projected desires and fantasies of significant others. However, the Symbolic Order and its laws and morals that channel the desires of the self and impart a form on it, by its very existence, initiate the possibility of transgressing that form.

As a result, sublimation is an inherently paradoxical challenge: on the one hand, it involves recognizing that forming ourselves is impossible without subjecting ourselves to the laws, norms, values and ideals imposed by society (the Imaginary and Symbolic). On the other hand, it propagates affirming and fueling a force (generated by the Real) that can never be completely appropriated by society and ourselves, and that can challenge and disrupt every possible social standard for self-formation and cultivation, which is a requirement for the emergence of novelty and the formation of a singular and healthy self. Any positive formulation of what this self should look like (like the ones formulated in health promotion) is inevitably bound to the Symbolic order, such that it is only in differentiating oneself from such formulations that any attempt at forming a singular self can take place. Empowerment as sublimation, then, consists in the very possibility of differentiation.

## Concluding remarks

The aim of this paper was to clarify in what sense health promotion can be said to empower individuals. We have started from the observation that most scholars maintain that empowerment crucially involves self-governance, but also showed that empowerment involves the capacity to adapt to societal and environmental norms. The advantage of our account of empowerment is that instead of trying to explain this inevitable tension away or to eliminate it, we take it as a starting-point for understanding what empowerment entails. Freud’s notion of sublimation is helpful in this regard because it highlights that self-governance implies the ability to re-direct instinctual and biological (sexual) drives to sociocultural acceptable goals, which highlights the tension between the two.

A reflection on Freud’s notion of sublimation revealed the difficulties arising when assessing empowerment independent of any social valuations, but also showed that it is equally impossible to make it dependent on social valuations alone. We proposed to understand this problematic in terms of a tension between the organism’s capacity to adapt to certain norms and its capacity of creating new norms. With Canguilhem and Lacan, we explored two ways in which this tension can be addressed.

Canguilhem’s account of health can be understood as providing an understanding of empowerment that is grounded in the ability to be able to tolerate the aggressions of a multiplicity of environments, implicating that self-governance consists in the ability to not merge completely with one particular environment. After all, just being able to adapt to one particular set of norms present in a particular environment undermines the ability to be normative in other environments. For Canguilhem, it is a biological given that it is impossible to think of individual and environment as separate entities, as well as that it is inevitable that individuals encounter a multiplicity of environments that each prompt a certain response. Therefore, even though that individuals continuously exercise their normativity, they evaluate their powers primarily in terms of their ability to adapt to the different environments that they face. However, we also saw that Canguilhem holds that the human must also adapt to how the environment is socially organized, without being determined by this particular organization. This implies that being empowered does not necessarily imply to live in accordance with specific societal norms, but consists in the ability of developing a critical relation to those.

Lacan places more emphasis on the critical integration of social norms. For Lacan, self-governance always involves incorporating projections, ideals and desires of significant others (the Imaginary), as well as the legal and moral construction of society as such (the Symbolic). Moreover, the regulative structures of the Imaginary and the Symbolic also neutralize the potential destructive influence of the drives on both society and the human self. Nevertheless, a complete coincidence with a particular projected norm or ideal is neither possible nor desirable, because there is always some-Thing (instigated by the Real) that escapes and frustrates any specific self-constitution; a stubborn residue is always left in the meshes of the symbolic order that does not fit and does not cooperate. For Lacan, recognizing and cultivating this residue is what makes sublimation possible. This Thing, as the mark of the Real, fuels the desire to transgress the symbolic boundaries set upon the subject without completely destroying them, which enables the formation of a singular self and sustaining of a social self. For Lacan, enduring and cultivating this tension is necessary for a healthy form of individuation.

The Lacanian perspective makes visible that empowerment involves the individual’s necessity to adapt to the environment and be shaped by it, as well as its desire to transgress the laws and ideals that this environment poses upon it. As a result, the very activity of self-organization is inevitability accompanied by the tension and struggle between the incorporation in the symbolic order in which someone is embedded and the ability to act against that order. This points to the impossibility of a completely autonomous mode of self-governance in the sense that people would be able to form themselves completely independent of the ideals imposed on them. Given that, according to Lacan, the subject neither completely coincides with itself, nor with the circumstances in which it finds itself, any particular organization can only be “successful” for a certain period. Therefore, the experience of being healthy is always at risk of being disturbed either by changes in the environment or by changes in the individual’s self-experience. In either case, the individual is continuously confronted with the necessity of imposing a new form onto itself, which fosters the desire for new forms of self-organization.

The dynamic understanding of the relationship between human being and environment brings us back to some aspects of Canguilhem’s account of health that are crucial for developing an apt understanding of empowerment. First, it is important to remain understanding empowerment as involving *bodily* experience. The experience of being healthy involves that someone is not exclusively focusing on one’s body, but instead is capable of using one’s body to transcend into other projects (Carel 2021). Experiencing one’s body as hindrance to engage in projects in the environment, is a mark of being unable to tolerate and cope with the aggressions of the environment (Toro et al. 2020). Second, Canguilhem helps reminding that the environment in which the organism lives is far from stable, both because the organism is inevitably in motion, constantly encountering new environments, and because any environment is partly constituted by the organism’s own normative capacities. This indicates that the desire for sublimation, and hence empowerment, is not only necessitated by the subject’s not coinciding with itself, but also by the multiplicity of environments it faces. Hence, from Canguilhem’s perspective, sublimation is not so much fueled by the desire to transgress the (symbolic) norms in a given environment. Rather, it consists of the ability of not-coinciding with a particular symbolic environment amongst the *multiplicity* of symbolic environments that someone inevitably encounters.

By integrating the findings of Freud, Canguilhem and Lacan, we can envisage a fruitful notion of empowerment as an ideal of health promotion. Since empowerment implies the ability to relate to a multiplicity of environments, it minimally entails to have the means to adapt to different environments without completely coinciding with one of them. This also implies that to be empowered is not to identify oneself with the realization of one specific ideal, such as one that is heavily informed by the discourse of (preventive) medicine. Building on Lacan, we suggested that the possibility of not coinciding with this ideal is made possible by the presence of a symbolic order in which it is integrated, because order always implies the possibility of transgression. Because of this, empowerment as a goal of health promotion should not consist merely in giving individuals control such that they can better align with a certain fixed ideal. Rather, empowerment should consist in the promotion of the ability to cope with both environmental changes and changing desires that evade complete control, which makes possible the formation of a singular self in the absence of a stable foundation.

## Footnotes

1 The boundaries between health promotion and disease prevention are not always clear. On the one hand it seems that health promotion is explicitly concerned with disease prevention since it crucially involves the idea that the promotion of health helps preventing the occurrence of future diseases. On the other hand, health promotion often establishes a close link between the promotion of health and the promotion of well-being, a connection that is less prevalent in the discourse of disease prevention. Hence, there are both significant overlaps and important differences between the two practices. A critical discussion of those is beyond the scope of this paper. We stick to the use of “health promotion,” as the notion of “empowerment” is most often discussed in this context.

2 Especially in his later works, Lacan extensively discusses the role and significance of the Real in different contexts under different and related phenomena and names, such as the Real, the Real Order, the Real Other, the unknowable x, the Thing, the gaze and the little object a. See Lacan (1997, pp. 43–87); Lacan (2006, pp. 671–703).
